# Influence of Light-Curing Time and Increment Thickness on the Properties of Bulk Fill Composite Resins With Distinct Application Systems

**DOI:** 10.1155/2024/2123406

**Published:** 2024-10-21

**Authors:** Carlos Rocha Gomes Torres, Taiana Paola Prado, Daniele Mara da Silva Ávila, Cesar Rogério Pucci, Alessandra Bühler Borges

**Affiliations:** Department of Restorative Dentistry, Institute of Science and Technology, Sao Paulo State University—UNESP, São José dos Campos, Brazil

**Keywords:** composite resins, degree of conversion, depth of cure; hardness; translucency

## Abstract

The objective of this study was to evaluate the influence of light-curing time and increment thickness on the microhardness and degree of conversion (DC) of bulk fill composite resins with different application systems. Translucency parameter (TP) was also measured. Specimens of resin composites were prepared in a circular matrix using a single increment with different thicknesses (2, 4, and 6 mm) and light-cured with distinct times (10, 20, and 40 s). The materials tested (*n* = 5 pergroup) were Filtek One (FO) bulk fill, Tetric N-Ceram (TC) bulk fill, SonicFill 3 (SF), VisCalor (VC) bulk. After 24 h, Knoop microhardness (KN) was measured, and the bottom/top ratio (B/T_ratio_) was calculated. The DC was measured using Fourier transformed infrared (FTIR) spectroscopy. The TP was assessed in additional specimens with 1 mm thick (*n* = 5). The data were statistically analyzed with analysis of variance (ANOVA) and Tukey's tests (5%). Significant differences were observed for all factors, for both B/T_ratio_ and DC (*p*  < 0.05). The higher increment thickness and the lower curing times resulted in lower B/T_ratio_ and DC means. The VC and TC resins exhibited the overall higher B/T_ratio_, and the highest TP. SF presented the lowest values of B/T_ratio_ and DC, with compromised polymerization at 6 mm depth. TP (means ± SD) were FO (12.85 ± 1.0)^1^, SF (15.62 ± 0.73)^2^, TC (20.32 ± 0.49)^3^, and VC (20.53 ± 0.73)^3^. We concluded that the greater the thickness of the increment, the lower the DC. Higher light curing times resulted on increased DC of the tested composites. The higher translucent materials VC and TC showed the greater B/T_ratio;_ and FO resin exhibited the higher DC values.

## 1. Introduction

Dental development professionals have been witnessing a notable evolution in the and improvement of resin-based restorative materials. The changes in the resin composites are mainly attributed to modifications in their filler and polymeric matrix [1]. In 2009, a category of methacrylate-based composite resins called “Bulk Fill” was introduced in the dental market [2].

Currently, the use of bulk fill composite resins for restoration of posterior teeth is widespread, with different options. They are presented as flowable or packable, according to the viscosity and handling characteristics. The compactable bulk fill resins can be extended up to the restoration surface due to their higher filler loads, which provide higher viscosity and facilitating the sculpting process [3]. Temporarily lowering the viscosity of the compactable resins during increment insertion represents an alternative to optimize its handling process while maintaining its mechanical properties [4]. Sonic energy can be applied while dispensing the material, thus producing a reduction in the viscosity during extrusion, facilitating the insertion procedure. After insertion of the resin into the cavity, the viscosity returns to its original level, allowing the conclusion of the restoration. The heating process has also been reported to be effective to reduce the materials viscosity, favoring its adaptation to the cavities walls and internal angles [5, 6]. Although any conventional resin composite material can be heated, a controlled mechanism and formulation was recently developed, using a thermoviscous technology designed to provide flow characteristics like those of the flowable materials [4].

The single increment insertion technique infers that the proper polymerization is a critical point, especially in deep cavities [7]. This technique involves placing a large volume of composite resin in a single layer, which can pose challenges for complete light penetration and effective polymerization throughout the material. Ensuring adequate polymerization is essential to prevent marginal deterioration, bacterial infiltration around the restoration, and minimize the risk of postoperative sensitivity [8, 9]. Although bulk-fill composites have been reported to be able to achieve adequate conversion of monomers into polymers, similar to conventional composites using incremental filling techniques [10–16], this has been shown to be highly material-dependent [3].Additionally, the final properties of the composites can be affected by the light-curing method [17, 18].

Seeking service efficiency, dentists have adopted shorter clinical steps to reduce clinical time expenses, which leveraged the use of bulk-fill resins but also led to a quest for reduced light-curing protocols [19]. Despite being fundamentally a chemical process, operators are responsible for controlling critical factors such as radiant energy, exposure time, and the positioning of the light tip to ensure satisfactory polymerization of the material [20]. This step must consider the amount of organic matrix, resin shade, and increment thickness [21]. With manufacturers offering a wide range of technical options, the decision-making process becomes more complex, requiring a deep understanding of material characteristics and their specific applications [22].

The aim of the present study was therefore to evaluate the influence of light-curing time and thickness increment on the microhardness of bulk-fill resins with different application techniques and cure mechanisms. Additionally, the analysis of the materials translucency was performed. The null hypotheses tested were (a) the type of bulk-fill material, the increment thickness, and the light-curing time did not influence the microhardness and degree of conversion (DC) results and (b) there is no difference of the translucency parameter (TP) between the tested materials.

## 2. Methods

### 2.1. Experimental Design

This study used resin composite specimens and followed a factorial 4 × 3 × 3 design, considering the experimental factors: composite resins (Table 1) at four levels (Filtek One (FO) bulk fill, Tetric N-Ceram (TC) bulk, SonicFill 3 (SF), and VisCalor (VC) bulk), increment thickness (2, 4, and 6 mm), and light-curing time (10, 20, and 40 s). The response variables were Knoop microhardness (KN) and DC. A total of 180 specimens were prepared (*n* = 5 for each final group). Additional specimens (*n* = 5 per material) were prepared for translucency analysis.

### 2.2. Specimens Preparation and Groups Division

Metallic matrices containing circular cavities were used to fabricate cylindric specimens with 5 mm of diameter and different thickness (2, 4, and 6 mm). The materials were applied inside the matrices in a single increment. A polyester matrix strip was placed under the matrix to produce a smooth and flat bottom surface. To prevent the formation of the oxygen-inhibited layer on the surface of the composite resin, a polyester matrix strip was placed over the resin. A glass coverslip was then applied to achieve a smoother and flatter surface [23].

The composites FO bulk fill and TC bulk fill were inserted inside the matrix by hand instruments. SF was applied using the SF handpiece, which produced sonic waves while the material was extruded from the capsule dispensing rate/speed was at “setting 3”. VC bulk was applied using the VC dispenser, which heated the material up to 60°C in 30 s. Over the surface of the uncured material, another polyester matrix strip was placed.

A single wave light-curing LED device (Demi, Kerr Dental, Orange, CA, EUA) with the wavelength 450–470 nm an emittance of 1.000 mW/cm^2^ was used to cure the specimens. The curing was performed for 10, 20, or 40 s, according to the group.

A small groove was performed on the surface of each specimen for later identification of the top side. For each combination of factors, five specimens were obtained (*n* = 5). All the specimen's preparation process was performed inside a temperature control chamber, kept at 37°C to standardize and simulate the body temperature. After that, the light-cured specimens were stored at 37°C for 24 h in the dark to allow the postcuring of the material.

### 2.3. Microhardness Analysis

The KN of the specimens was measured using a microhardness tester (FM-700, Future Tech, Tokyo, Japan), 10 g load for 10 s. Three indentations were performed on top and bottom surfaces, and the mean for each side was calculated. The bottom/top microhardness ratio (B/T_ratio_) [24] was calculated for each specimen and the number obtained was multiplied by 100, obtaining the percentage of the bottom side hardness in relation to the top side (100%), using the following formula:

  $$\begin{array}{c} \text{B}/{\text{T}}_{\text{ratio}}\%=\left({\text{KN}}_{\text{bottom}}/{\text{ KN}}_{\text{top}}\right)\times 100. \end{array}$$

### 2.4. DC Analysis

The DC was measured using a Fourier-transform infrared (FTIR) spectrometer (Frontier, PerkinElmer, Waltham, MA, USA) with an attenuated total reflectance (ATR) accessory (five measures). The spectrum ranged between 1800–1400 cm^−1^ and the FTIR spectra were recorded with 10 scans at a resolution of 4 cm^−1^. The uncured composites were placed directly on the ATR crystal using a silicone mold. The bottom surfaces of the cured specimens were placed over the crystal, and the readings were performed. The spectra obtained were normalized using the spectrometer's software (Spectrum, PerkinElmer, Waltham, MA, USA).

The peak height ratio of the absorbance intensities of methacrylate carbon double bond peak at 1637 cm^−1^ and that of an internal standard peak (IS) at 1608 cm^−1^ (aromatic carbon double bond) was calculated for VC, SF, and TC. For FO the IS peak was replaced by 1600 cm^−1^ due to the absence of the 1608 cm^−1^ peak. The percentage of remaining unreacted double bonds was determined by the DC, which was calculated by the ratio variation between the cured and uncured composite according to the following formula:

  $$\begin{array}{c} \text{DC}=\left[1\text{–}\left(1634{\text{ cm}}^{-1}/\text{IS}\right)\text{ peak after curing}/\left(1634{\text{ cm}}^{-1}/\text{IS}\right)\text{ peak before curing}\right]\times 100. \end{array}$$

### 2.5. Translucency Analysis

For analysis of the TP, 20 additional specimens were prepared (*n* = 5 per group). The specimens were made using a matrix that contained circular cavities measuring 5 mm in diameter and 1 mm deep. Polyester matrix strip was placed under the matrix to produce specimens with smooth and flat bottom surfaces. The evaluated composite resins were applied inside the cavities according to the manufacturer's recommendations as previously described in the specimen preparation section. A glass slide was used to press the material and create a flat surface parallel to the matrix surface and was then removed. A monowave LED curing device (Demi, Kerr Dental, Orange, CA, USA) with an emittance of 1000 mW/cm^2^ of blue light was used to polymerize the samples. The wavelength range was 450–470 nm. The light tip was positioned in the center of the specimen in direct contact with the mylar strip for 20 s.

The polyester matrix strip was removed, and the specimens were then placed in Eppendorf tubes, which were identified according to the resin brand and specimen number. Then, the specimens were stored at 37°C for 24 h in the dark, to allow postpolymerization of the material.

The specimens were positioned over a white and a black standard background and analyzed using a colorimetric spectrophotometer (CM2600d, Konica Minolta, Osaka, Japan) and CIELAB system. The device was set for small area view (SAV), D65 standard light source with 100% UV included, specular component included (SCI), and observer angle at 2°. The TP was calculated according to the following formula:

  $$\begin{array}{c} {\text{TP}}_{ab}={\left[{\left({\text{L}}_{{\text{⁣}}^{*}B}\text{–}{\text{ L}}_{{\text{⁣}}^{*}W}\right)}^{2}+{\left({\text{a}}_{{\text{⁣}}^{*}B}\text{–}{\text{ a}}_{{\text{⁣}}^{*}W}\right)}^{2}+{\left({\text{b}}_{{\text{⁣}}^{*}B}\text{–}{\text{ b}}_{{\text{⁣}}^{*}W}\right)}^{2}\right]}^{1/2}. \end{array}$$

### 2.6. Statistical Analysis

The data of KN (top and bottom), B/T_ratio_, and DC were analyzed using three-way analysis of variance (ANOVA) (kind composite x cavity depth x curing time) and Tukey's test. For the TP, one-way ANOVA was used. The software Statistica for windows (StatSoft, Tulsa, OK, USA) was used and the significance level of 5% was adopted.

## 3. Result

The three-way ANOVA statistical test showed significant differences between the composite resins tested for the microhardness analyses and DC (*p*  < 0.001). For the top microhardness, significant differences were observed only for the type of composite and the curing time (*p*  < 0.001). The increment thickness did not show significant effect (*p* = 0.976). For the bottom microhardness, significant differences were observed for all factors both for B/T_ratio_ and DC (*p*  < 0.001).

The tables refer to the results of the three-factor ANOVA for each isolated factor, as there was a significant difference for each factor. To facilitate the visualization of the distinct materials under each tested condition, we presented the detailed data in the figures.

Table 2 shows the results of Tukey's test for the composite factor. VC and FO showed similar results of top microhardness, higher than the other materials. For the bottom microhardness, all materials were different between them, and the higher means were observed for VC. For the B/T_ratio_, VC showed the highest values, statistically similar to TC. SF showed the smaller values of bottom microhardness and B/T_ratio_, as well as DC. FO showed the higher values of DC, followed by VC.

Table 3 shows the results of Tukey's test for the factor increment thickness. For the bottom microhardness, B/T_ratio_, and DC, significantly smaller means were observed for the deeper cavities.

Table 4 shows the results of Tukey's test for the factor curing time. For all analyses, the increase of curing time significantly increased the means.

**Figure 1:**
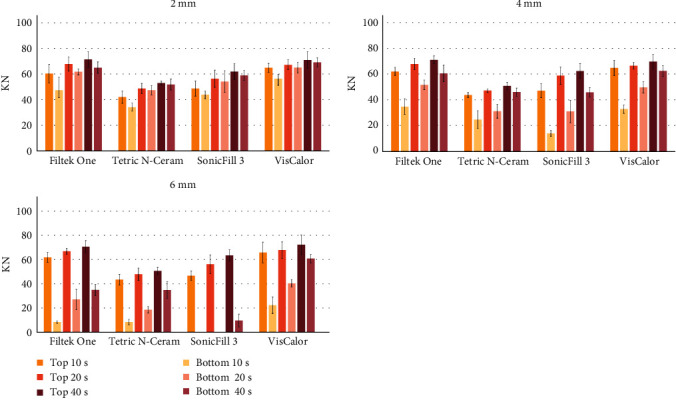
Microhardness means (SD) of the top and bottom sides for all composites, curing times, in 2, 4, and 6 mm thickness increments. SD, standard deviation.

Figure 1 shows the absolute top and bottom microhardness values for all composites, for the increment thickness of 2, 4, and 6 mm, respectively. It can be clearly seen that the increased curing time increases the microhardness of the material. It must be highlighted that SF at the depth of 6 mm for the curing time of 10 and 20 s presented insufficient curing. It can also be seen that the higher increment thickness increased the differences between the top and bottom microhardness values.

**Figure 2:**
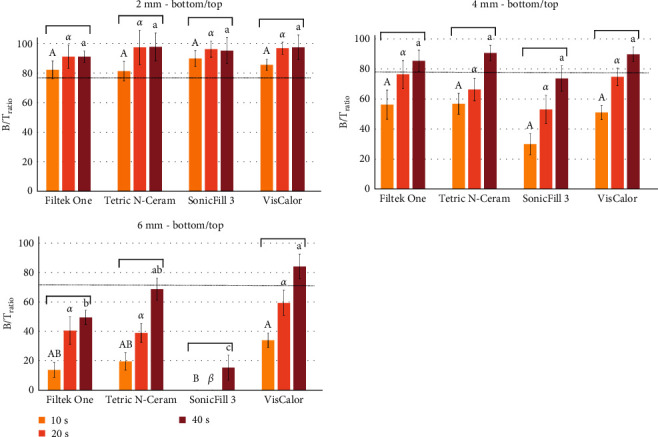
Means (SD) of B/T_ratio_ for all composites, curing times, in 2, 4, and 6 mm thickness increments. Different letters mean significant differences for the materials within time (capital letters for 10 s, greek letters for 20 s, and lowercase letters for 40 s). The horizontal bars combine similar conditions for the same material in different times. Dotted line represents the conventional 80% B/T_ratio_. B/T_ratio_, bottom/top microhardness ratio; SD, standard deviation.

Figure 2 shows the B/T_ratio_ of all composites, for the increment thicknesses of 2, 4, and 6 mm, respectively. The conventional 80% B/T_ratio_ was achieved for all materials and times in 2 mm specimens. In 4 mm specimens, it was achieved by FO, TC, and VC composites only after 40 s of light-curing, and in 6 mm specimens, only VC achieved this ratio after 40 s of light curing. For the 2 mm specimens, no significant differences were observed for the light-curing times. However, for the deeper cavities, the increase of the curing time significantly increased the B/T _ratio_, with significantly higher values when the resin was light-cured for 40 s compared to 10 s. For 6 mm depth, VC and TC showed the higher B/T_ratio_ when light-cured for 40 s.

**Figure 3:**
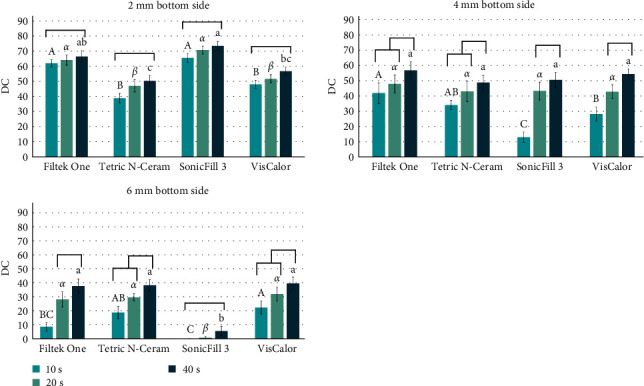
Means (SD) of DC for bottom for all composites, curing times, in 2, 4, and 6 mm thickness increments. Different letters mean significant differences for the materials within time (capital letters for 10 s, greek letters for 20 s, and lowercase letters for 40 s). The horizontal bars combine similar conditions for the same material in different times. DC, degree of conversion; SD, standard deviation.

Figure 3 shows the DC at the bottom of the specimens for 2, 4, and 6 mm depth, respectively. FO and SF showed the overall higher DC means in 2 mm specimens, for the different light-curing times. No differences were observed between the light-curing times for the same material (horizontal bars). For the 4 mm specimens, significant differences between the materials were observed only for 10 s, with intermediate DC means for VC and lower values for SF. Increasing the light-curing time from 10 s to 40 s resulted in significantly higher DC for all materials. For the 6 mm specimens, higher values of DC were observed with VC and TC for 10 s, but for 20 s and 40 s, similar results were obtained, except for SF, which showed DC values near zero. As observed with the 4 mm specimens, materials light-cured for 40 s showed significantly higher DC compared to 10 s, while 20 s produced intermediate values.

**Figure 4:**
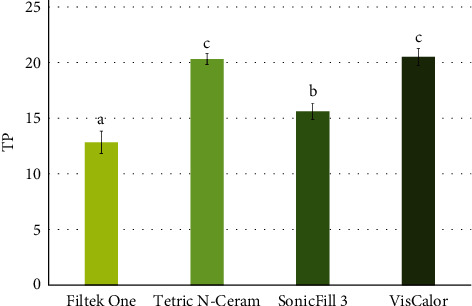
Means (SD) of TP for all composite tested and results of Tukey's test. Groups followed by the same letter do not show significant differences. SD, standard deviation; TP, translucency parameter.

The one-way ANOVA test showed significant differences between the groups for the TP (*p* = 0.0001).  Figure 4 shows the means of TP and results of Tukey's test for all groups. FO was significantly less translucent than all the others, while VC and TC were similar and the most translucent materials evaluated.

## 4. Discussion

The findings of this study reinforced the evidence that microhardness and DC decreased with the increase of specimen thickness and reduction of photoactivation time, as expected [25, 26]. Furthermore, these parameters varied according to the materials tested. So, the first tested null hypothesis was rejected.

Although the comparison between the microhardness values of the different materials is important to assess their mechanical properties, the absolute values should not be used to directly compare the polymerization potential of composites with differing filler type, size, or loading [24]. The top hardness values in our study did not reveal significant differences in the three thicknesses. For the bottom hardness values, all materials decreased as the thickness of the samples increased, which is expected, since the light source was applied on the top surfaces of all materials. Thus, to better reflect the polymerization efficacy, the B/T_ratio_ was calculated and compared to the arbitrary cutoff score of 80% as an indirect measurement of polymerization [24, 27, 28]. Additionally, the FTIR was used as a direct assessment [3], since both techniques present adequate correlation when evaluated using the same type of material [24, 29].

Obtaining a sufficient DC that results in higher KN, modulus of elasticity, and color stability of composite resins [30] still represents a challenge for bulk-fill composite resins [3]. In our study, the three tested variables (thickness of the increment, light-curing time, and the type of resin) influenced the DC.

It is believed that increasing the light-curing time or using a higher-power light source helps to provide sufficient energy to the bottom of the restoration [31, 32], however, there is still no consensus on the absolute value of energy required to obtain an ideal polymerization since this value depends on the characteristics of each composite resin [3]. For the 2 mm increments, all the materials achieved the critical value of 80% for B/T_ratio_. For the 4 mm increments, this value was obtained only when the increments were light-cured for 40 s, except for SF that did not exhibit an adequate polymerization in this thickness even using a high-power light source (1000 mW/cm^2^). For the 6 mm increments, satisfactory results were observed only for VC resin and with an extended polymerization time (40 s). Nevertheless, using the thickness of 4 mm, all materials achieved an adequate DC when light cured for the recommended time, except SF. This corroborates previous studies that report attenuated polymerization of SF resins at a 4 mm thickness [25, 33].

Differences in the performance between the resins tested may be related to several factors, with these mechanisms being almost unique to each manufacturer [3, 34–36]. To ensure proper mechanical and curing properties, chemical modifications were made to the type and amount of monomers [37–39], change in the proportion between the filler and the organic matrix [40, 41].

All resins investigated have combinations of high molecular weight polymers, such as Bis-GMA (540 g/mol) and Bis-EMA (629 g/mol), responsible for promoting the reduction of contraction stress by reducing the number of reactive groups in the matrix; and low molecular weight, such as triethylene glycol dimethacrylate (TEGDMA; 286.32 g/mol), responsible for controlling the rigidity of the developing and final polymeric matrix [42]. With the objective of modulating the polymerization shrinkage, FO and TC resins additionally have monomers such as addition fragmentation monomer (AFM) and urethane dimethacrylate (UDMA) in their organic matrix, respectively, which due to incorporation of photoactive groups, allow a greater flexible connection during polymer network formation thus achieving a high DC and mesh density [40, 42]. These modifications in the resin's organic matrix lead to positive clinical outcomes. A clinical trial revealed that after 1 year, restorations employing FO resin exhibited superior marginal adaptation compared to those made with another bulk-fill resin that lacked an additional system to manage polymerization shrinkage [43].

Enhancing light penetration efficiency is another strategy utilized to mitigate the risk of marginal gap formation caused by internal stress in inadequately polymerized structures. Certain composite resins have increased the size of the inorganic fillers to reduce the filler/matrix interface, thereby minimizing light scattering within the material and facilitating a higher DC [44, 45], however, this increases the translucency of the composite resin [3]. These data corroborate our findings, where the TC and VC resins, which exhibited a higher DC, demonstrated the highest translucency values, consistent with results reported in another study [46]. Consequently, the second null hypothesis was also rejected, as differences were observed between the materials.

Another alternative with the same objective, used by TC, is the addition of photoinitiators as intensifiers of the conventional process with camphorquinone, which influence the polymerization kinetics of the material. Ivoclar Vivadent resins have a photoinitiation enhancer called Ivocerin [38, 39]. This initiator is activated by ultraviolet light (380–450 nm), and generates more efficient free radicals than camphorquinone, which leads to greater photopolymerizable activity with high monomer conversion, due to its greater absorption of visible light, providing deeper polymerization. This photoinitiator also influences the polymerization kinetics of the material, providing the material with less stress generation by 60%–70%, making the material excellent for its purpose to increase polymerization even with large increments [38–40]. A 5-year clinical study demonstrated the practical outcomes of the modifications in the TC resin composition. Restorations performed using the bulk-fill technique showed performance similar to those performed using the incremental filling technique, with both techniques exhibiting similar survival rates and low annual failure rates. This highlights the reliability and durability of these resins in clinical practice [47].

The importance of using a polywave light-curing device has been emphasized by the manufacturer when using composite resins that have different photoinitiators. However, studies show that there is no difference in the microhardness and DC values obtained with mono or polywave light-curing device [12, 48–50].

Knowing that increased conversion generally equates to better properties of polymeric materials and that higher temperatures promote faster and greater conversion [5, 51], a composite resin was specially developed with viscosity modulated by heating. This material uses a thermoviscous technology that involves a specific organic matrix and surface treatment of the inorganic matrix [4]. In fact, VC results of B/T_ratio_ and DC were among the highest values observed in this study, demonstrating the efficacy of the preheating process and corroborating with the results obtained with preheating conventional composite resins [52]. However, such gain could only be guaranteed when sufficient irradiance was applied, as also reported previously [33, 46, 53]. However, we emphasize that the interpretation of the data presented here should be made with caution, as clinical performance is determined by various factors related to the operator, the material, the patient, and the tooth. Only long-term clinical trials can reveal the performance of materials over time.

## 5. Conclusion

Within the limitations of this in vitro study, it can be concluded that:

• The increase of curing time improved the microhardness and DC of the materials tested. The deeper the cavity, the smaller the bottom microhardness and the DC values;
• A curing time of 40 s is recommended to ensure better light curing efficacy of bulk-fill resins at a thickness of 4 mm;
• The heated resin (VC) was one of the most translucent materials resulting in a polymerized material with an overall higher bottom microhardness and the B/T_ratio_;
• The increase in thickness (6 mm) of SF severely compromised the light curing at the bottom.

## Figures and Tables

**Table 1:** Specifications of the materials tested.

Composite resin	Composition		Inorganic particle size	Increment thickness/curing time indicated by the manufacturer (>1.000 mW/cm^2^)	Shade/lot
	Organic matrix	Inorganic matrix (% by weight)			
FO bulk fill restorative 3M/ESPE (St. Paul, USA)	AFM, AUDMA, UDMA, DDDMA	Zirconia filler, silica filler, ytterbium trifuoride, zirconia/silica nano agglomerates, pigments, camphorquinone 76.5%	Nano hybrid	4 mm/20 s	A2E/NA17808

TC bulk fill Ivoclar Vivadent (Schaan, Liechtenstein)	Bis-GMA, Bis-EMA, UDMA	Standard filler barium/aluminum/silica glass, prepolymer, ytterbium trifluoride, mixed oxides, additives, stabilizers, pigments, acyl phosphine oxide, camphorquinone, ivocerin 75%–77%	Hybrid	4 mm/10 s	IV A/X27163

SF Kerr (Biberach, Germany)	Bis-GMA Bis-EMA, TEGDMA, EBPDMA, MPS	Barium glass, oxide, chemicals, silicon dioxide, ytterbium trifluoride, pigments, camphorquinone 84%	Nano hybrid	5 mm/10 s*⁣*^*∗*^	A2/7010825

VC bulk Voco (Cuxhaven, Germany)	Bis-GMA, Aliphatic dimethacrylates	Glass fillers, ytterbium trifluoride, pigments, camphorquinone 83%	Nano hybrid	4 mm/20 s	A2/2019121

*Note*: The (*⁣*^*∗*^) indicates that Kerr recommends additional 10 s cures on both the buccal and lingual surfaces of the tooth.

Abbreviations: AFM, addition fragmentation monomer; AUDMA, aromatic urethane dimethacrylate; Bis-EMA, bisphenol A ethoxylate dimethacrylate; Bis-GMA, bisphenol-A diglycidil ether dimethacrylate; DDDMA, 1,12-dodecane dimethacrylate; EBPDMA, ethoxylated bisphenol-adimethacrylate; FO, Filtek One; MPS, 3-(trimethoxysilyl) propyl methacrylate; SF, SonicFill 3; TC, Tetric N-Ceram; TEGDMA, triethylene glycol dimethacrylate; UDMA, urethane dimethacrylate; VC, VisCalor.

**Table 2:** Means (SD) KN and DC values for the type of composite factor.

Composite	Top KN*⁣*^*∗*^	Bottom KN*⁣*^*∗*^	B/T_ratio_*⁣*^*∗*^	DC*⁣*^*∗*^
TC	47.55 (4.75)^a^	33.08 (14.80)^c^	68.83 (28.81)^ab^	38.76 (10.47)^c^
SF	55.76 (8.35)^b^	27.95 (22.38)^d^	49.6 (39.60)^c^	35.85 (29.92)^d^
FO	66.64 (5.83)^c^	43.79 (18.63)^b^	65.15 (26.50)^b^	45.96 (19.12)^a^
VC	67.78 (6.31)^c^	50.94 (16.39)^a^	75.01 (23.51)^a^	41.76 (12.11)^b^

Abbreviations: B/T_ratio_, bottom/top microhardness ratio; DC, degree of conversion; FO, Filtek One; KN, Knoop microhardness; SD, standard deviation; SF, SonicFill 3; TC, Tetric N-Ceram; VC, VisCalor.

*⁣* ^*∗*^Groups followed by the same letter in the columns do not have significant differences.

**Table 3:** Means (SD) KN and DC values for the cavity increment thickness.

Increment thickness (mm)	Top KN*⁣*^*∗*^	Bottom KN*⁣*^*∗*^	B/T_ratio_*⁣*^*∗*^	DC*⁣*^*∗*^
2	59.53 (10.51)^a^	54.71 (10.71)^a^	91.94 (10.32)^a^	57.85 (10.82)^a^
4	59.32 (10.21)^a^	40.42 (15.33)^b^	67.25 (21.67)^b^	42.13 (12.98)^b^
6	59.46 (10.93)^a^	22.14 (18.60)^c^	35.46 (27.75)^c^	21.77 (14.94)^c^

Abbreviations: B/T_ratio_, bottom/top microhardness ratio; DC, degree of conversion; KN, Knoop microhardness; SD, standard deviation.

*⁣* ^*∗*^Groups followed by the same letter in the columns do not have significant differences.

**Table 4:** Means (SD) KN and DC values for curing time factor.

Curing time (s)	Top KN*⁣*^*∗*^	Bottom KN*⁣*^*∗*^	B/T_ratio_*⁣*^*∗*^	DC*⁣*^*∗*^
10	54.31 (10.4)^a^	27.43 (17.52)^a^	50.17 (30.94)^a^	31.77 (20.13)^a^
20	59.95 (9.37)^b^	39.88 (19.60)^b^	66.14 (31.39)^b^	41.77 (18.21)^b^
40	64.03 (9.48)^c^	49.88 (17.02)^c^	78.11 (25.17)^c^	48.21 (17.25)^c^

Abbreviations: B/T_ratio,_ bottom/top microhardness ratio; DC, degree of conversion; KN, Knoop microhardness; SD, standard deviation.

*⁣* ^*∗*^Groups followed by the same letter in the columns do not have significant differences.

## Data Availability

The data supporting the findings of this study have been compiled in Excel, stored in the ICT UNESP repository, and are available from the corresponding author upon request.
